# Impact of patient and treatment characteristics on glycemic control and hypoglycemia in patients with type 2 diabetes initiated to insulin glargine or NPH: A post hoc, pooled, patient-level analysis of 6 randomized controlled trials

**DOI:** 10.1097/MD.0000000000006022

**Published:** 2017-02-03

**Authors:** Francesca Porcellati, Jay Lin, Paola Lucidi, Geremia B. Bolli, Carmine G. Fanelli

**Affiliations:** a Perugia University School of Medicine, Department of Medicine, Perugia, Italy; b Novosys Health, Flemington, NJ.

**Keywords:** basal insulin, insulin glargine, meta-analysis, NPH insulin, obesity, type 2 diabetes

## Abstract

**Background:**

The goal of this post hoc analysis was to determine key patient and treatment-related factors impacting glycosylated hemoglobin (A1C) and hypoglycemia in patients with uncontrolled type 2 diabetes who were initiated to basal insulin (neutral protamine Hagedorn [NPH] or glargine).

**Methods:**

Using individual patient-level data pooled from 6 treat-to-target trials, 2600 patients with type 2 diabetes on oral antidiabetic agents initiated to insulin glargine or NPH and treated for 24 to 36 weeks were analyzed.

**Results:**

Both treatments led to significant reduction in A1C levels compared with baseline, with no differences between treatment groups (mean ± standard deviation; glargine: −1.32 ± 1.2% vs NPH: −1.26 ± 1.2%; *P* = 0.15), with greater reduction in the BMI ≥30 kg/m^2^ group than in the BMI <30 kg/m^2^ group. Glargine reduced A1C significantly more than NPH in the BMI <30 kg/m^2^ group (−1.30 ± 1.18% vs −1.14 ± 1.22, respectively; *P* = 0.008), but not in the BMI ≥ 30 kg/m^2^ group (−1.37 ± 1.19 vs −1.48 ± 1.22, respectively; *P* = 0.18). Similar proportions of patients achieved A1C target of <7% (glargine 30.6%, NPH 29.1%; *P* = 0.39). Incidence of severe and severe nocturnal hypoglycemia was significantly lower in glargine versus NPH-treated patients (2.0% vs 3.9%; *P* = 0.04, and 0.7% vs 2.1%; *P* = 0.002, respectively), and occurred primarily in the BMI <30 kg/m^2^ group.

**Conclusions:**

Initiation of basal insulin is highly effective in lowering A1C after oral antidiabetic agent failure. Glargine decreases A1C more than NPH in nonobese patients, and reduces the risk for severe and severe nocturnal hypoglycemia versus NPH both in obese and nonobese patients, but more so in nonobese patients. Thus, it is the nonobese patients who may benefit more from initiation of basal insulin as glargine than NPH.

## Abstract 2

Supplemental Digital Content is available in the text

## 1 Introduction

In patients with type 2 diabetes, the treatment goal is to reduce glycemic levels to prevent the development and progression of microvascular and macrovascular complications that may significantly increase disease burden and morbidity/mortality.

The American Diabetes Association and the European Association for the Study of Diabetes have outlined therapeutic steps for optimal treatment of type 2 diabetes, suggesting that treatment needs to begin or be adjusted when glycosylated hemoglobin (A1C) is ≥7.0% (≥53 mmol/mol), with an ultimate treatment target of <7.0% (<53 mmol/mol).^[1]^ Recommended management considers basal insulin as an essential component of the treatment strategy, and timely initiation of insulin (as basal), when lifestyle changes and metformin therapy fail to control A1C <7.0% (<53 mmol/mol), is an option.^[1,2]^

The timely initiation of basal insulin has a number of advantages, as indicated by the Outcome Reduction with an Initial Glargine Intervention study.^[3]^ First, timely initiation produces a dose-dependent reduction in fasting blood glucose (FBG).^[4]^ Second, basal insulin may be easily titrated, tailoring the dose for individual patients based upon FBG levels.^[1–4]^ Lastly, it is usually well-tolerated, with few adverse effects (only modest risk for hypoglycemia and increase in body weight), and is safe.^[3,4]^ For many years, the intermediate-acting neutral protamine Hagedorn (NPH) insulin has been the most common insulin treatment for patients with type 2 diabetes. Although NPH is effective at helping patients achieve glycemic control, it is also associated with a significant increase in the risk for hypoglycemia, due to unwanted plasma insulin peak that occurs 4 to 6 hours after injection.^[5]^ As time progressed, newer, longer-acting insulins with smoother activity were developed to better reproduce the physiology of basal insulin.^[6,7]^ Insulin glargine is one of these longer-acting basal insulins, and clinical trials have repeatedly shown noninferiority of glargine in terms of efficacy as compared with NPH, though with significantly fewer hypoglycemia events. A previous meta-analysis^[8]^ examined a large number of clinical trials, however, based on different methodologies of insulin treatment.

The present analysis examined pooled, individual, patient-level data from 6 multisite, randomized clinical trials with similar research methodologies. The objective of this observation was to compare the efficacy and safety of glargine and NPH added to oral therapy in patients with type 2 diabetes, using pooled patient-level data from 6 treat-to-target clinical trials.^[9–14]^ Study endpoints included reduction in A1C, proportion of patients achieving A1C target, and incidence of severe and severe nocturnal hypoglycemia. In addition, the patient-level pooled data were also used to assess the impact of patient- and treatment-related factors on A1C reduction and hypoglycemia incidence. Previous research suggests that a higher body mass index (BMI) might more heavily impact the declining ß-cell function^[15]^ and blunt the pharmacodynamics of basal insulin,^[16]^ thus attenuating the efficacy of insulin treatment^[17]^; therefore, data were also stratified by BMI.

## 2 Research design and methods

### 2.1 Description of data

Individual data from patients with type 2 diabetes were pooled and analyzed, akin to similar recent analyses,^[18,19]^ from the intent-to-treat populations of 6 multinational, multicenter, randomized, open-label clinical trials with similar research methodologies,^[9–14]^ comparing treatment with either insulin glargine or NPH insulin (Supplemental Table 1).

### 2.2 Patient selection in the analyzed randomized clinical trials

Patients with type 2 diabetes were enrolled in each of their respective studies, if inadequately controlled on 1 or 2 oral agents (sulfonylureas, metformin, pioglitazone, or rosiglitazone) for ≥6 months and insulin-naïve. Patients included within the analysis were aged 20 to 80 years, had a BMI between 20 and 40 kg/m^2^, and had A1C levels between 7.5% (58 mmol/mol) and 12% (108 mmol/mol). Patients were excluded if they were nightshift workers, pregnant, or breastfeeding; had a history of ketoacidosis, self-reported inability to recognize hypoglycemia, or had a history of alcohol or drug abuse; were treated with insulin or any investigational drugs within the previous 3 months; or had any clinically relevant somatic or mental disease. All pooled trials were conducted in accordance with the Declaration of Helsinki and approved by the independent ethics committee or institutional review board of each center or site. Before participating in any study-related procedure, all patients provided written informed consent.

### 2.3 Study designs of the analyzed randomized clinical trials

For all 6 trials analyzed, the primary objective was to assess the efficacy and safety of glargine compared with NPH in patients with type 2 diabetes. Patients were treated daily for 24,^[10–13]^ 28,^[9]^ or 36^[14]^ weeks, according to study criteria. In addition, patients either continued their previous oral agents therapy,^[9,13,14]^ or this was substituted with 3 or 4 mg of glimepiride.^[10–12]^ Across studies, there were some differences in the insulin starting dose and in the dose titration method used. Studies either calculated the initial dose as a function of each patient's FBG level,^[10,12,14]^ or patients were started on the same preset dose.^[9,11,13]^ Across all studies, dose was titrated to achieve a target FBG level, which ranged from 4.4 to 5.5 mmol/L. In 4 studies, a predefined regimen was used to titrate dose.^[10–13]^ In the remaining 2 studies, titration was at the discretion of the investigator, or a function of the patient's condition or laboratory measurements.^[9,14]^

### 2.4 Efficacy outcome measures

In 5 of the studies that were part of the pooled data, the primary efficacy outcome was change in A1C level from baseline to endpoint.^[9–12,14]^ In the remaining study, the primary outcome was the percentage of patients achieving A1C ≤7.0% (≤53 mmol/mol) without a single episode of symptomatic nocturnal and/or severe hypoglycemia.^[13]^

### 2.5 Safety outcome measures

Severe hypoglycemia was defined as symptoms consistent with hypoglycemia during which the patient required the assistance of another person and associated with either a glucose level of <3.1 mmol/L^[11,13]^ or <2.8 mmol/L,^[9,10,12,14]^ or prompt recovery after oral carbohydrate, intravenous glucose, or glucagon.

Nocturnal hypoglycemia was defined as hypoglycemia that occurred while the patient was asleep, after an evening insulin injection and before rising in the morning. Additional adverse events (AEs) were also recorded within all pooled studies.

### 2.6 Statistical analysis

For unadjusted data analysis, categorical variables were analyzed by the chi-square test and continuous variables by independent 2-sample Student *t* test. A *P* value of <0.05 was regarded as statistically significant. To further confirm the results of the individual pooled data analyses, meta-analysis with random-effect model was also utilized to analyze A1C change.

Multivariable generalized linear regressions were used to evaluate the impact of treatment on A1C change and likelihood of severe hypoglycemia and severe nocturnal hypoglycemia, after controlling for key covariates that may potentially influence the outcomes. Covariates included insulin type, age, baseline BMI, duration of diabetes, baseline A1C and insulin dose, and metformin/sulfonylurea usage. Meta-analytic analyses were carried out in Review Manager 5.1 (Cochrane Collaboration, Copenhagen, Denmark). Other statistical analyses were carried out in SAS 9.3 (SAS Institute, Cary, NC).

## 3 Results

### 3.1 Baseline characteristics

Individual data from a total of 2600 patients were pooled from the 6 studies (glargine: n = 1385, NPH: n = 1215). Patients were stratified according to BMI (kg/m^2^) into <30 and ≥30 cohorts; overall, similar proportions of patients were represented in the 2 BMI cohorts for glargine and NPH (BMI <30: 65% and 66%, BMI ≥30: 35% and 34%, respectively).

No significant differences were observed across the 2 treatment arms in age, weight, BMI, type 2 diabetes disease duration, or A1C levels (Supplemental Table 2). The percentage of patients treated with sulfonylurea before insulin initiation among the 2 groups treated with glargine or NPH was also similar (77.6% vs 74.7%, respectively). When patients were considered according to BMI stratification, the BMI <30 cohort was significantly older than the BMI ≥30 cohort (*P* < 0.001), had a higher percentage of patients on sulfonylureas (83.3% vs 63.0%, respectively; *P* < 0.001), had higher baseline A1C levels (*P* < 0.001), and longer disease duration (*P* < 0.001). Within both BMI cohorts, a comparison of baseline characteristics between glargine and NPH treatment groups revealed no significant differences.

### 3.2 Effect on glycosylated hemoglobin

**Figure 1:**
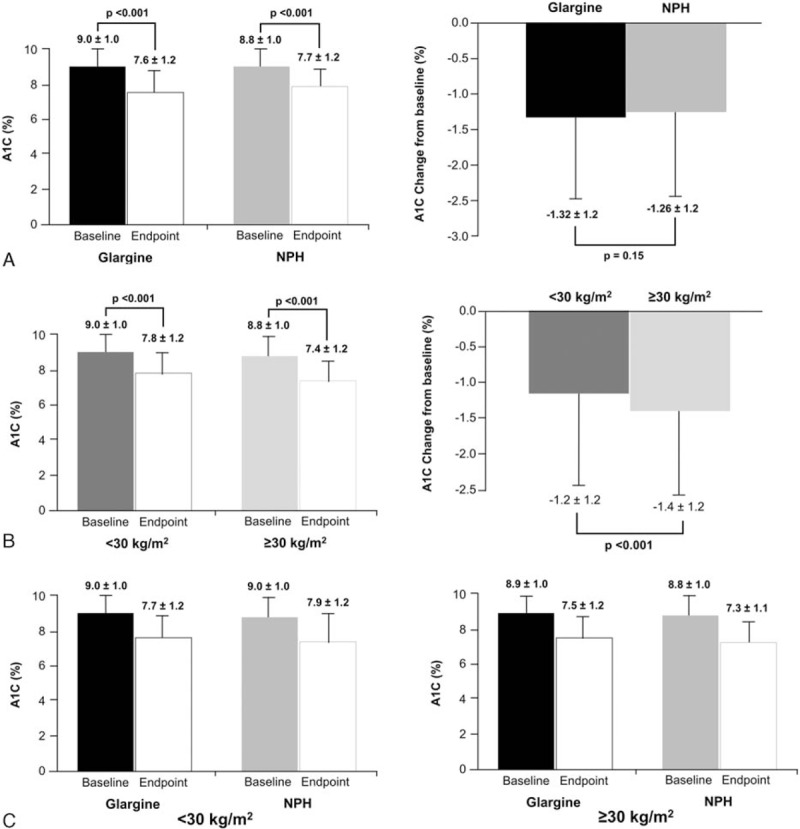
A1C levels at baseline and endpoint by treatment group (A), BMI group (B), and treatment/BMI (C). A1C, glycosylated hemoglobin; BMI, body mass index.

At study endpoint in patients treated with either glargine or NPH, no significant differences in A1C were revealed between treatment arms (Fig. 1). Patients in both the glargine and NPH groups had significant reductions in A1C from baseline to study endpoint (−1.3 ± 1.2% vs −1.26 ± 1.2%, respectively) with no differences between the glargine and NPH-treated patients.

Baseline and endpoint A1C levels were significantly lower in those with BMI ≥30 compared with BMI <30 (Fig. 1). Patients with BMI ≥30 experienced a significantly greater change in A1C from baseline than those with BMI <30 (−1.4% vs −1.2%, respectively; *P* < 0.001). Significant between-treatment difference in A1C was measured in favor of glargine treatment over NPH in the BMI <30 group (−1.3% vs −1.1%, respectively; *P* = 0.008); the comparison was not significant in the BMI ≥30 group.

There was no significant difference in the proportion of patients achieving target A1C of <7% (<53 mmol/mol) between the glargine and NPH-treated groups (30.6% vs 29.1%, respectively). Between BMI groups, a significantly higher percentage of patients with BMI ≥30 achieved target A1C than with BMI <30 (38.9% vs 25.0%, respectively; *P* < 0.001). For those with BMI <30, a significantly greater proportion of patients treated with glargine than NPH achieved the target (27.0% vs 22.8%, respectively; *P* = 0.047); however, this comparison was not significant in the BMI ≥30 group.

### 3.3 Effect on FBG

At baseline, patients randomized in the glargine group had significantly higher mean ± standard deviation FBG than NPH in all comparison cohorts (overall: 204.1 ± 54.2 vs 196.2 ± 55.2 mg/dL, respectively; *P* = 0.003; BMI <30: 207.7 ± 55.2 vs 200.1 ± 57.7, respectively; *P* = 0.006; BMI ≥30: 197.4 ± 51.7 vs 188.8 ± 49.5, respectively; *P* = 0.01). Though change from baseline comparisons were not adjusted for baseline FBG, patients treated with glargine had significantly greater reductions in FBG from baseline than NPH overall (−79.4 ± 59.0 vs −69.1 ± 59.8 mg/dL, respectively; *P* < 0.001). FBG reductions were significantly greater with glargine than NPH in patients with BMI <30 (−85.4 ± 58.9 vs −71.7 ± 62.9, respectively; *P* < 0.001); however, these reductions were not significantly different in those with BMI ≥30 (−68.4 ± 57.7 vs −64.0 ± 53.3, respectively; *P* = 0.24).

### 3.4 Effect on hypoglycemia

**Figure 2:**
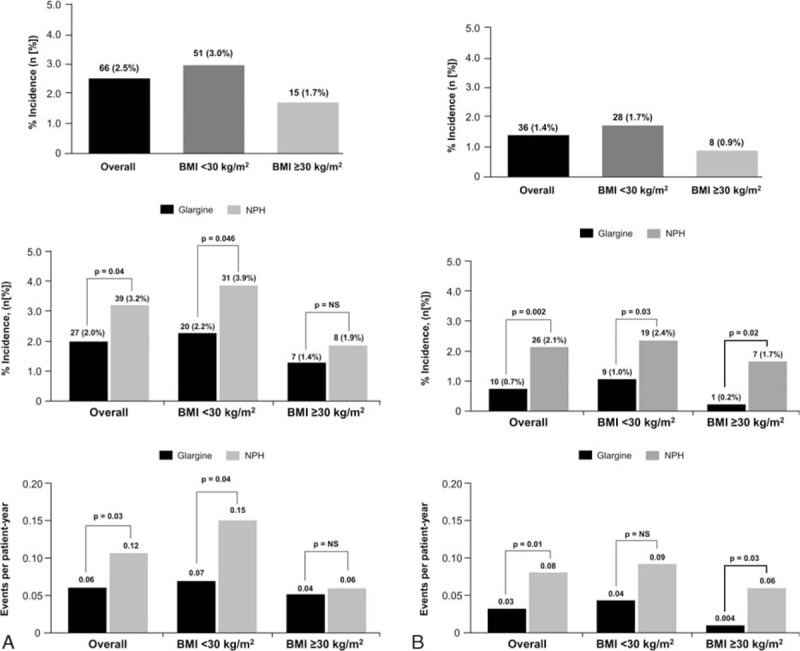
Incidence and events per patient-year of severe (A) and severe nocturnal (B) hypoglycemia by treatment group and BMI. BMI, body mass index.

Overall, the number of patients reporting severe hypoglycemia was small; however, the incidence of both severe and severe nocturnal hypoglycemia was significantly greater in NPH than glargine-treated patients (*P* = 0.04 and *P* = 0.002, respectively) (Fig. 2). A significantly greater number of severe and severe nocturnal hypoglycemia events per patient-year occurred in the NPH group than the glargine group (severe: 0.12 ± 0.95 vs 0.06 ± 0.48, respectively; *P* = 0.03; severe nocturnal: 0.08 ± 0.73 vs 0.03 ± 0.37, respectively; *P* = 0.01). Patients with BMI <30, compared with those with BMI ≥30, experienced greater incidence of severe hypoglycemia (3.0% vs 1.7%, respectively) and severe nocturnal hypoglycemia (1.7% vs 0.9%, respectively).

Treatment comparisons within BMI groups found that NPH-treated patients experienced significantly greater incidence of severe hypoglycemia than glargine-treated patients in the BMI <30 group (3.9% vs 2.2%, respectively; *P* = 0.046), but not in those with BMI ≥30 (1.9% vs 1.4%; *P* = NS). However, NPH-treated patients had higher incidence of severe nocturnal hypoglycemia than glargine in both BMI groups (BMI <30: 2.4% vs 1.0%, respectively; *P* = 0.03; BMI ≥30: 1.7% vs 0.2%, respectively; *P* = 0.02). Severe hypoglycemia events per patient-year were more frequent with NPH as compared with glargine in those with BMI <30 (0.15 vs 0.07, respectively; *P* = 0.04) and BMI ≥30 (0.06 vs 0.04, respectively; *P* = NS). This was also true for severe nocturnal hypoglycemia (BMI <30: 0.09 vs 0.04, respectively; *P* = NS; BMI ≥30: 0.06 vs 0.004, respectively; *P* = 0.03).

### 3.5 Achieving A1C target with no severe hypoglycemia

No significant differences were observed between glargine and NPH for the proportion of patients reaching target A1C levels of <7% (53 mmol/mol) without incidence of severe hypoglycemia (29.8% vs 28.4%, respectively). This was also true for both the BMI <30 (25.9% vs 22.0%, respectively) and BMI ≥30 (37.0% vs 40.7%, respectively) groups.

### 3.6 Insulin dose

Insulin dose at study endpoint was also analyzed between treatment arms and within BMI cohorts. An overall comparison at study endpoint revealed that insulin dose (U/kg) for the glargine-treated group was significantly higher than for the NPH group (0.44 vs 0.41 U/kg, respectively; *P* = 0.003). The within-BMI comparison found that for patients with BMI <30, insulin dose was not significantly different (glargine: 0.39 U/kg vs NPH: 0.37 U/kg; *P* = 0.07); however, for those with BMI ≥30, glargine-treated patients had a significantly higher endpoint insulin dose (glargine: 0.51 U/kg vs NPH: 0.47 U/kg; *P* = 0.017).

### 3.7 Meta-analytic method results

**Figure 3:**
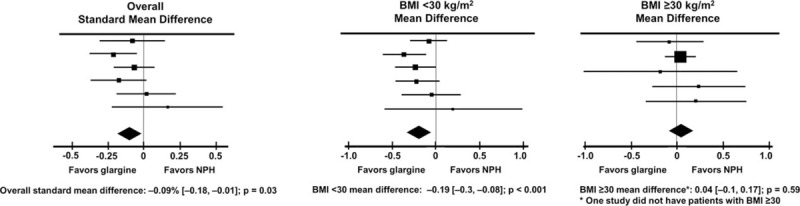
Meta-analysis of studies: A1C change by treatment. A1C, glycosylated hemoglobin.

Heterogeneity of the 6 trials utilized in this analysis was assessed using Q statistics from the Mantel–Haenszel method; for the 6 trials, the *P* value was 0.81 for A1C change, 0.87 for severe hypoglycemia, and 0.56 for severe nocturnal hypoglycemia, confirming homogeneity with respect to these variables among the selected trials. Meta-analytic data analysis with a random-effects model showed that treatment with glargine versus NPH was associated with a greater reduction in A1C (standardized mean difference: −0.09%, confidence interval [CI] −0.18 to −0.01, *P* = 0.03). This was largely driven by the greater reduction in A1C for glargine-treated patients in the BMI <30 group (mean difference: −0.19%, 95% CI −0.3 to −008, *P* < 0.001); no significant treatment difference was observed in the BMI ≥30 group (mean difference: +0.04%, 95% CI −0.10 to 0.17, *P* = NS) (Fig. 3).

### 3.8 Multivariate regression analysis

#### 3.8.1 Change in A1C

After controlling for key patient characteristics, including insulin type, age, baseline BMI, duration of diabetes, baseline HbA1c, initial insulin dose, and metformin and SU usage, multivariate regression was performed to analyze patient-related and treatment characteristics associated with A1C change for each BMI group. Within the BMI <30 group, treatment with glargine (*P* < 0.0001), female sex (*P* < 0.001), and baseline A1C (*P* < 0.0001) were strongly associated with a greater A1C reduction (Table 1). For those with BMI ≥30, only baseline A1C (*P* < 0.001) and insulin dose (*P* < 0.03), and not insulin type, were associated with greater A1C reduction (Table 1).

**Table 1:**
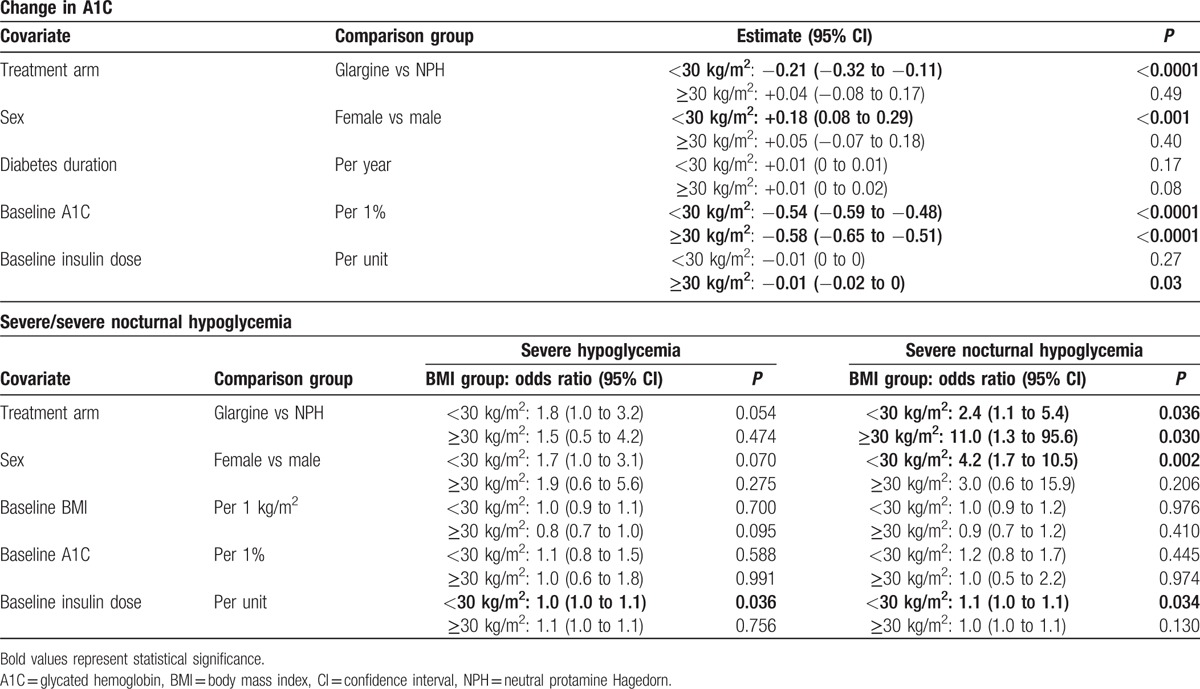
Impact of covariates.

#### 3.8.2 Hypoglycemia

Multivariate regression was also performed to analyze the impact of covariates on the incidence of severe and severe nocturnal hypoglycemia for each BMI group. Initial insulin dose was associated with a higher risk of severe hypoglycemia in those with BMI <30, whereas no covariates were identified with higher risk in the ≥30 group (Table 1). With respect to severe nocturnal hypoglycemia, both NPH treatment and initial insulin dose were associated with higher risk of severe nocturnal hypoglycemia in the BMI <30 group, whereas only NPH treatment was associated with a higher risk in the BMI ≥30 group (Table 1).

## 4 Discussion

This post hoc analysis confirms and expands previous knowledge about outcomes of basal insulin initiation in type 2 diabetes insufficiently controlled on oral glucose-lowering drugs. What was known already is that basal insulin is efficacious in lowering A1C close to the target of 7.0% (53 mmol/mol), regardless of whether insulin glargine or NPH is used. It was also known that glargine, as compared with NPH, reduces the risk of hypoglycemia.

What is new in this post hoc pooled analysis is that: in people with BMI ≥30, A1C decreases more than in people with BMI <30 (regardless of NPH or glargine); glargine is superior to NPH in lowering A1C in people with BMI <30; severe and nocturnal severe hypoglycemia shows greater incidence in people with BMI <30 as compared with those with BMI ≥30; and glargine lowers the risk of severe and nocturnal severe hypoglycemia in both groups, but more so in people with BMI <30. This new information may be relevant to personalized treatment of patients with type 2 diabetes.^[1]^

The fact that basal insulin, regardless of NPH or glargine, decreases A1C more in obese than in nonobese patients with type 2 diabetes (independent of baseline A1C) might be explained by greater endogenous insulin secretion, which is reported in the former as compared with latter patients.^[20]^ It is likely that the lower A1C in the obese patients was explained by less postprandial hyperglycemia as a consequence of more preserved insulin secretion in this group. According to this hypothesis, leaner people with longer diabetes duration are not only more insulin-deficient, but also remain more insulin-deficient at mealtime after supplementation of basal insulin, as compared with patients with BMI ≥30.

This interpretation is supported by the second new finding of the present study, that glargine is superior to NPH in lowering A1C in nonobese, but not obese, patients, as indicated by our analysis (Fig. 3). The pharmacokinetic/pharmacodynamic superiority of glargine versus NPH^[5,6]^ translated into lower fasting plasma glucose and, therefore, lower A1C. This occurred in nonobese people with more deficient endogenous insulin secretion, not in those with insulin still secreted in large quantities, as in obesity.

Our analysis supports the concept that risk for hypoglycemia is greater in nonobese than in obese patients, regardless of type of basal insulin used.^[21,22]^ The previous hypothesis about greater insulin deficiency and longer diabetes duration (both well-recognized risk factors for hypoglycemia)^[21,22]^ may well account for this finding. However, glargine reduces hypoglycemia risk, as compared with NPH (severe and nocturnal severe hypoglycemia), both as incidence and also cumulative events over time. Notably, only severe hypoglycemia episodes were analyzed in the present study to have a solid database and limit the margins of uncertainty about definition and documentation of the more frequent nonsevere hypoglycemia.^[1]^ As expected in the populations examined of insulin-naïve patients initiating basal insulin, severe (and nocturnal severe) hypoglycemia was quite infrequent. Yet, despite the low incidence and low event rates over time, it was nevertheless possible to document superiority of glargine versus NPH in reducing the hypoglycemia episodes. The risk reduction was more consistent in the subgroup with more versus fewer hypoglycemia events, that is, in nonobese as compared with obese patients. Thus, glargine reduces the risk of severe and nocturnal severe hypoglycemia more in the subgroup of nonobese people where the hypoglycemia risk is greater. Notably, this patient-level pooled analysis indicates that in nonobese patients, glargine reduces A1C more and at the same time reduces the risk for severe hypoglycemia more, as compared with NPH. As far as we know, these are new findings.

These findings may be relevant to the present recommendation of personalizing treatment in type 2 diabetes (with the final aim of lowering A1C while minimizing hypoglycemia). In this context, it is worth mentioning the recent observation from the ORIGIN trial, in which several clinical characteristics were described to be associated with greater hypoglycemia risk than others.^[23]^ Glargine may be a preferred option compared with NPH, primarily in nonobese patients with BMI <30, where it results in lower A1C, and, at the same time, lower risk for severe and nocturnal severe hypoglycemia. In obese patients, there is no evident benefit of glargine in terms of A1C lowering, but there is less nocturnal severe hypoglycemia with glargine, though severe hypoglycemia is no different as compared with NPH. However, as time goes by and insulin treatment is prolonged over the years, the risk of hypoglycemia increases, and a basal insulin such as glargine may reduce the hypoglycemia risk as compared with NPH, even in this subgroup of obese people.

As a final note, in theory, the greater use of sulfonylurea in people with BMI <30 (83.3%) as compared with those with BMI >30 (63%) may have contributed to the higher risk for hypoglycemia in the former versus latter group. However, the multivariate regression analysis indicated that the differential use of sulfonylurea was not associated with a greater incidence of severe and nocturnal severe hypoglycemia in the former group. Instead, it is the BMI per se, and its interaction with the initial insulin dose, which accounts for the differences in hypoglycemia observed between the 2 treatment groups.

Limitations of this post hoc analysis include the fact that only evening, not morning, dosing of basal insulin effects has been examined. Glargine in the morning may exhibit quite different pharmacodynamics effects as compared with evening.^[24]^ In addition, only severe and nocturnal severe episodes of hypoglycemia were analyzed. However, the strength of this study is the numerosity obtained through the pooling of patient-level data in multiple randomized clinical trials which examined varieties of people with type 2 diabetes (reflective of the heterogeneity of the disease).

## 5 Conclusions

Initiation of basal insulin is highly effective in lowering A1C after OAD failure. Glargine decreases A1C more than NPH in nonobese patients, and reduces the risk for severe and severe nocturnal hypoglycemia versus NPH both in obese and nonobese patients, but more so in nonobese patients. Thus, nonobese patients may benefit more from initiation of basal insulin as glargine than as NPH.

## Supplementary Material

Supplemental Digital Content
