# Supplementary material for: Impact of patient and treatment characteristics on glycemic control and hypoglycemia in patients with type 2 diabetes initiated to insulin glargine or NPH: A post hoc, pooled, patient-level analysis of 6 randomized controlled trials
Source: Medicine (Baltimore). 2017 Feb 3;96(5):e6022. doi: 10.1097/MD.0000000000006022 (PMC5293468; doi:10.1097/MD.0000000000006022)
Supplement: Supplemental Digital Content [file medi-96-e6022-s001.doc]

**SUPPLEMENTAL TABLE 1. Trial Characteristics**

| **Study**  **(Treatment Duration)** | **Treatment  Arm** | **Treatment** | **Primary Analysis** |
| --- | --- | --- | --- |
| **Pan et al.^9^ (24 weeks) LEAD/4012 Study** | NPH (n = 223) | Pre-study: OADs only  Study: 3 mg glimepiride QD for 4 wks, then added GLA or NPH, QD | Demonstrate noninferiority of baseline to endpoint Δ in A1C between GLA and NPH |
|  | GLA (n = 220) |  |  |
| **Eliaschewitz et al.^10^**  **(24 weeks) HOE901/4013 LA Study** | NPH (n = 250) | Pre-study: OADs only  Study: 4 mg glimepiride for 4 wks, then added GLA or NPH, QD | Δ in A1C from baseline to the end of the study |
|  | GLA (n = 231) |  |  |
| **Yki-Jarvinen et al.^11^**  **(36 weeks)**  **LANMET/6001 Study** | NPH (n = 49) | Pre-study: SU + MET or MET alone  Study: GLA or NPH, QD, added to current MET dosage (SU discontinued where used) | Δ in A1C from baseline to endpoint |
|  | GLA (n = 61) |  |  |
| **Fritsche et al.^12^ (24 weeks) 4001 Study** | NPH (n = 232) | Pre-study: OADs only  Study: 3 mg glimepiride QD for 4 wks, then added GLA or NPH, QD | Δ in A1C from baseline to endpoint  Frequency of patients experiencing hypoglycemic episodes |
|  | GLA, am (n = 236) |  |  |
|  | GLA, pm (n = 227) |  |  |
| **Riddle et al.^13^**  **(24 weeks) Treat-to-Target/4002 Study** | NPH (n = 389) | Pre-study: OADs only  Study: GLA or NPH, QD, added to previous OAD regimen | Percentage of patients achieving A1C ≤7% without symptomatic nocturnal and/or severe hypoglycemia |
|  | GLA (n = 367) |  |  |
| **Kawamori et al.^14^**  **(28 weeks) 3102 Study** | NPH (n = 168) | Pre-study: SU + α-glycosidase inhibitor and/or biguanide  Study: GLA or NPH, QD, added to previous treatment | Δ in A1C from start of treatment to completion of treatment |
|  | GLA (n = 167) |  |  |

NPH = neutral protamine hagedorn, GLA = glargine, OAD = oral anti-diabetes drug, QD = once daily, A1C = glycated hemoglobin, SU = sulfonylurea, MET = metformin.

**SUPPLEMENTAL TABLE 2.** Demographic and Baseline Characteristics

|  | **Glargine**  **(n = 1385)** | **NPH**  **(n = 1215)** | ***P* Value** | **BMI <30 kg/m^2^**  **(n = 1693)** | **BMI ≥30 kg/m^2^**  **(n = 907)** | ***P V*alue** |
| --- | --- | --- | --- | --- | --- | --- |
| **Age (yr)** | 57.0 (9.4) | 57.4 (8.6) | 0.22 | 58.0 (9.0) | 55.7 (9.4) | < 0.001 |
| **Gender, male, n (%)** | 728 (52.6%) | 621 (51.1%) | 0.49 | 922 (54.5%) | 427 (47.1%) | 0.71 |
| **Weight (kg)** | 79.0 (19.1) | 78.4 (19.5) | 0.40 | 69.1 (12.9) | 96.6 (16.2) | < 0.001 |
| **BMI (kg/m^2^)** | 28.3 (5.0) | 28.4 (5.2) | 0.87 | 25.3 (2.9) | 34.0 (3.2) | < 0.001 |
| **T2DM duration (yr)** | 9.8 (6.4) | 10.2 (6.0) | 0.19 | 10.7 (6.3) | 8.6 (5.8) | < 0.001 |
| **A1C (%)** | 9.0 (1.0) | 9.0 (1.0) | 0.93 | 9.0 (1.0) | 8.8 (1.0) | < 0.001 |
| **Taking sulfonylureas, n (%)** | 1074 (77.6%) | 908 (74.7%) | 0.09 | 1411 (83.3%) | 571 (63.0%) | < 0.001 |

All values are mean (standard deviation) unless otherwise noted.

A1C: glycated hemoglobin; BMI: body mass index; NPH: neutral protamine Hagedorn; T2DM: Type 2 diabetes mellitus
